# Phytochemicals and biological studies of plants from the genus *Balanophora*

**DOI:** 10.1186/1752-153X-6-79

**Published:** 2012-08-01

**Authors:** Xiaohong Wang, Zizhen Liu, Wenlin Qiao, Ruiyang Cheng, Bin Liu, Gaimei She

**Affiliations:** 1 Department of Traditional Chinese Medicine, School of Chinese Pharmacy, Beijing University of Chinese Medicine, Beijing, 100102, China

## Abstract

This review focus on the phytochemical progress and biological studies of plants from the genus *Balanophora* (Balanophoraceae) over the past few decades, in which most plants growth in tropical and subtropical regions of Asia and Oceania, and nearly 20 species ranged in southwest China. These dioeciously parasitic plants are normally growing on the roots of the evergreen broadleaf trees, especially in the family of Leguminosae, Ericaceae, Urticaceae, and Fagaceae. The plants are mainly used for clearing away heat and toxic, neutralizing the effect of alcoholic drinks, and as a tonic for the treatment of hemorrhoids, stomachache and hemoptysis. And it has been used widely throughtout local area by Chinese people.

Cinnamic acid derivative tannins, possessing a phenylacrylic acid derivative (e. g. caffeoyl, coumaroyl, feruloyl or cinnamoyl), which connected to the C(1) position of a glucosyl unit by *O*-glycosidic bond, are the characteristic components in genus *Balanophora*. In addition, several galloyl, caffeoyl and hexahydroxydiphenoyl esters of dihydrochalcone glucosides are found in *B*. *tobiracola*, *B*. *harlandii*, and *B*. *papuana*. Other compounds like phenylpropanoids, flavonoids, terpenoids and sterols are also existed. And their biological activities, such as radical scavenging activities, HIV inhibiting effects, and hypoglycemic effects are highlighted in the review.

## Review

## Introduction

*Balanophora* is a genus belonging to the family Balanophoraceae which possesses about 120 species all over the world. Many of them distribute in tropical and subtropical regions of Asia and Oceania, and nearly 20 species are widely ranged in southwest of China [1,2]. These types of dioeciously parasitic plants are normally growing on the roots of the evergreen broadleaf trees, especially in the family of Leguminosae, Ericaceae, Urticaceae, and Fagaceae [1,3-5]. Species in this genus have miscellaneous biological properties such as clearing away heat and toxic, neutralizing the effect of alcoholic drinks, and used as a tonic for the treatment of hemorrhoids, stomachache and hemoptysis by local people in China. The plants of genus *Balanophora* have been recorded in the Compendium of Materia Medica and were called ‘*Gecaihua*’ or ‘*Geru*’ [3,5,6].

Phytochemical investigation of genus *Balanophora* showed that the hydrolysable tannins are rich in species, particularly the ellagitannins. Structurally, cinnamic acid derivative tannins are the chemically-characterised constituents. It possesses a phenylacrylic acid derivative (e. g. caffeoyl, coumaroyl, feruloyl or cinnamoyl), which connected to the C(1) position of a glucosyl unit by *O*-glycosidic bond, and part of galloyl, caffeoyl and hexahydroxydiphenoyl (HHDP) are linked with the C(1), C(3), C(4) and / or C(6) positions through aromatic ester bonds. 1-*O*-caffeoyl-(4-*O*-galloyl)-β-D-glucopyranose (**2**) and 1-*O*-caffeoyl-(6-*O*-galloyl)-β-D-glucopyranose (**3**), which were first reported from *B*. *harlandii* in 2000 by Teng *et al*., have the analogous feature [7]. In addition, several galloyl, caffeoyl and hexahydroxydiphenoyl esters of dihydrochalcone glucosides (43–51) found in *B*. *tobiracolaB*. *harlandii*, and *B*. *papuana*, displayed obviously radical-scavenging activities, inhibiting HIV, as well as hypoglycemic effects, *etc*. [1,3,6,8-10]. Aside from those, the galloyl, HHDP and their derivatives linking the glucosyl unit at C(1), C(3), C(4) and / or C(6) positions through ester bonds are major constituents in the genus *Balanophora*[1,2,11-18].

In this review, we mainly summarize the study progress of phytochemical over the past decades and list the entire chemical compounds isolated from the genus *Balanophora*. Meanwhile, the biological activities of the plant extracts and chemical constituents are highlighted as well.

### The phytochemical studies of genus *Balanophora*

Phytochemical Constituents. – There are several sorts of chemical constituents existing in genus *Balanophora*. The reported diverse chemical structures of tannins substances owe to their functional groups and the attended modes as well as linked locations of its substituting groups. Extensive studies have led to the identification of many other compounds, such as C_6_-C_3_ and C_6_-C_3_-C_6_ constituents, terpenoids, sterols and so forth. The structures of compounds in the Figure [see Additional file 1] and their names are numbered 1–149, the corresponding plant sources and references are listed in the Table 1.

**Table 1:** Chemical constituents from genus balanophora

**No.**	**Name**	**Source**	**Ref.**
**1**	1-*O*-(*E*)-caffeoyl-3-*O*-galloyl-β-D-glucopyranose	*B*. *harlandii*, *B*. *spicata*, *B*. *japonica*, *B*. *fungosa*, *B*. *laxiflora*	[1,8,14,16,19,20]
**2**	1-*O*-caffeoyl-(4-*O*-galloyl)-β-D-glucopyranose	*B*. *harlandii*, *B*. *japonica*	[7,14]
**3**	1-*O*-caffeoyl-(6-*O*-galloyl)-β-D-glucopyranose	*B*. *harlandii*, *B*. *japonica*	[7,14]
**4**	1-*O*-(*E*)-caffeoyl-4,6-(*S*)-HHDP-β-D-glucopyranose	*B*. *japonica*, *B*. *fungosa*, *B*. *laxiflora*	[14,16,20]
**5**	1,2-di-*O*-(*E*)-caffeoyl-β-D-glucopyranose	*B*. *japonica*	[14]
**6**	1,3-di-*O*-(*E*)-caffeoyl-β-D-glucopyranose	*B*. *japonica*	[14]
**7**	1-*O*-(*E*)-caffeoyl-3,4-di-*O*-galloyl-β-D-glucopyranose	*B*. *japonica*	[14]
**8**	1-*O*-(*E*)-caffeoyl-4,6-di-*O*-galloyl*-β*-D-glucopyranose	*B*. *laxiflora, B*. *japonica, B*. *polyandra*	[5,14,15]
**9**	1-*O*-(*E*)-caffeoyl-3-*O*-galloyl-4,6-(*S*)-HHDP-β-D-glucopyranose	*B*. *laxiflora*, *B*. *japonica*, *B*. *fungosa*	[5,14,16,19]
**10**	1,3-di-*O*-(*E*)-caffeoyl-4,6-(*S*)-HHDP-β-D-glucopyranose	*B*. *laxiflora*, *B*. *japonica*	[5,14]
**11**	1,3-di-*O*-caffeoyl-4-*O*-galloyl-β-D-glucopyranose	*B*. *japonica*	[14,17]
**12**	1-*O*-(*E*)-caffeoyl-3,4,6-tri-galloyl-β-D-glucopyranose	*B*. *fungosa*	[16,19]
**13**	1,2,6-tri-*O*-caffeoyl-β-D-glucopyranose	*B*. *japonicaB*. *laxiflora*	[14,17]
**14**	1,3-di-*O*-galloyl-4,6-(*S*)-HHDP-*β*-D-glucopyranose	*B*. *laxiflora*, *B*. *polyandra*, *B*. *japonica*	[5,13-15,20]
**15**	1,3-di-*O*-galloyl-β-D-glucopyranose	*B*. *harlandii*, *B*. *laxiflora*, *B*. *japonica*, *B*. *polyandra*	[1,5,14,15]
**16**	1-*O*-galloyl-β-D-glucopyranose	*B*. *polyandra*	[15]
**17**	1,3,4-tri-*O*-galloyl-β-D-glucopyranose	*B*. *harlandii*, *B*. *japonica*	[1,14]
**18**	3-*O*-(*E*)-caffeoyl-1-*O*-galloyl-β-D-glucopyranose	*B*. *polyandra*	[15]
**19**	1,2,6-tri-*O*-galloyl-β-D-glucopyranose	*B*. *polyandra*	[15]
**20**	1,4-di- *O*-galloyl-β-D-glucopyranose	*B*. *japonica*	[14]
**21**	1,2,4-tri- *O*-galloyl-β-D-glucopyranose	*B*. *japonica*	[14]
**22**	1,2,6-tri-*O*-galloyl-β-D-glucopyranose	*B*. *laxiflora*, *B*. *japonica*	[5,14]
**23**	1,4,6-tri-*O*-galloyl-β-D-glucopyranose	*B*. *japonica*	[14]
**24**	1,3,4,6-tetra-*O*-galloyl-β-D-glucopyranose	*B*. *japonica*	[14]
**25**	1-*O*-galloyl-4,6-[(*S*)-HHDP]-β-D-glucopyranose	*B*. *japonica*, *B*. *polyandra*	[14,15]
**26**	3-*O*-(*E*)-caffeoyl-1,4-di-*O*-galloyl-β-D-glucopyranose	*B*. *japonica*	[14]
**27**	6-*O*-(*E*)-caffeoyl-1,3,4-tri-*O*-galloyl-β-D-glucopyranose	*B*. *japonica*	[14]
**28**	1-*O*-[(*E*)-caffeoyl]-4,6-[1,1'-(3,3',4,4'-tetrahydroxydibenzofurandicarboxyl)]-β-D-glucopyranose	*B*. *polyandra*, *B*. *japonica*	[15,21]
**29**	balanophotannins A	*B*. *japonica*	[21]
**30**	balanophotannins C	*B*. *japonica*	[21]
**31**	1-*O*-(*E*)-caffeoyl-6-*O*-(*S*)-brevifolincarboxyl-β-D-glucopyranose	*B*. *japonica*	[22]
**32**	1,3-di-*O*-galloyl-6-*O*-(*S*)-brevifolincarboxyl-β-D-glucopyranose	*B*. *japonica*	[22]
**33**	balanophotannins F	*B*. *japonica*	[22]
**34**	balanophotannins G	*B*. *japonica*	[22]
**35**	balapolyphorin A	*B*. *polyandra*	[15]
**36**	1-*O*-(*E*)-cinnamoyl-3-galloyl-4,6-(*S*)-HHDP-β-D-glucopyranose	*B*. *fungosa*	[16]
**37**	1-*O*-(*E*)-cinnamoyl-4-galloyl-β-D-glucopyranose	*B*. *fungosa*	[16]
**38**	1-*O*-(*E*)-coumaroyl-4,6-(*S*)-HHDP-β-D-glucopyranose	*B*. *japonica*, *B*. *fungosa*	[14,16]
**39**	1-*O*-(*E*)-coumaroyl-3-galloyl-4,6-(*S*)-HHDP-β-D-glucopyranose	*B*. *fungosa*	[16]
**40**	1-*O*-(*E*)-coumaroyl-3,4,6-tri-galloyl-β-D-glucopyranose	*B*. *fungosa*	[16]
**41**	1-*O*-(*E*)-*p*-coumaroyl-3-*O*-galloyl-β-D-glucopyranose	*B*. *harlandii*	[1]
**42**	balapolyphorin B	*B*. *polyandra*	[15]
**43**	3-hydroxyphloretin 4'-*O*-(6''-*O*-galloyl)-β-D-glucoside	*B*. *harlandii*, *B*. *tobiracola*	[1,8]
**44**	3-hydroxyphloretin 4'-*O*-(3'',4''-di-*O*-galloyl)-β-D-glucoside	*B*. *tobiracola*	[8]
**45**	3-hydroxyphloretin 4'-*O*-(4'',6''-di-*O*-galloyl)-β-D-glucoside	*B*. *tobiracola*	[8]
**46**	3-hydroxyphloretin 4'-[4'',6''-di-*O*-(*S*)-HHDP-β-D-glucoside]	*B*. *harlandii*, *B*. *tobiracola*	[1,8]
**47**	3-hydroxyphloretin 4'-*O*-[3''-*O*-galloyl-4'', 6''-*O*-(*S*)-HHDP]-β-D-glucoside	*B*. *tobiracolaB*. *harlandii*	[1]
**48**	3-hydroxyphloretin 4'-*O*-[3''-*O*-caffeoyl-4'', 6''-*O*-(*S*)-HHDP]-β-D-glucoside	*B*. *tobiracola*	[8]
**49**	phloretin 4'-*O*-[3'-*O*-galloyl-4',6'-*O*-(*S*)-HHDP]-β-D-glucoside	*B*. *tobiracola*	[8]
**50**	papuabalanols A	*B*. *papuana*	[9]
**51**	papuabalanols B	*B*. *papuana*	[9]
**52**	3-*O*-galloyl-4,6-[(*S*)-HHDP]-β-D-glucopyranose	*B*. *japonica*, *B*. *polyandra*	[14,15]
**53**	3-*O*-galloyl-β-D-glucopyranose	*B*. *harlandii*, *B*. *laxiflora*, *B*. *japonica*, *B*. *polyandra*	[1,5,14,15]
**54**	6-*O*-galloyl-β-D-glucopyranose	*B*. *laxiflora*	[5]
**55**	3-*O*-[(*E*)-caffeoyl]-4-*O*-galloyl-β-D-glucopyranose	*B*. *japonica*	[14]
**56**	6-*O*-(*E*)-caffeoyl-β-D-glucopyranose	*B*. *japonica*	[14]
**57**	2,6-di-*O*-galloyl-D-glucopyranose	*B*. *japonica*	[14]
**58**	3,4,6-tri-*O*-galloyl-D-glucopyranose	*B*. *japonica*	[14]
**59**	gallic acid	*B*. *laxiflora*, *B*. *harlandii*, *B*. *polyandra*, *B*. *simaoensis*, *B*. *japonica B*. *fungosa*	[5,7,15,23,24]
**60**	methyl gallate	*B*. *polyandra*	[15]
**61**	ellagic acid	*B*. *simaoensis*	[23]
**62**	methyl *p*-cumarate	*B*. *japonica*	[25]
**63**	coumaric acid	*B*. *laxiflora*, *B*. *fungosa*, *B*. *abbreviata*	[5,16,26]
**64**	*p*-hydroxycinnamic-β-D-glucopyranose	*B*. *fungosa*	[16]
**65**	caffeic acid	*B*. *laxiflora*, *B*. *simaoensis*, *B*. *japonica*	[5,23-25]
**66**	caffeic acid methyl ester	*B*. *japonica*	[25]
**67**	caffeic acid ethyl ester	*B*. *spicata*, *B*. *Japonica*	[19,25]
**68**	methylconiferin	*B*. *polyandra, B*. *simaoensis, B*. *involucrata*	[15,27-29]
**69**	butylconiferin	*B*. *simaoensis*	[28]
**70**	coniferin (= 4-(3-hydroxyprop-1-en-1-yl)-2-methoxyphenyl-β-D-glucopyranoside)	*B*. *harlandii*, *B*. *laxiflora*, *B*. *polyandra*, *B*. *polyandra*, *B*. *fungosa*, *B*. *involucrata*, *B*. *japonica*, *B*. *abbreviata*, *B*. *involucrata*	[1,5,15,16,18][25-27,29]
**71**	coniferin aldehyde-β-D-glucoside	*B*. *latisepala*	[30]
**72**	4-*O*-(6'-*O*-*p*-counmaroyl-β-D-glucopyranosyl)-coniferyl aldehyde	*B*. *latisepala*	[30]
**73**	ferulyl aldehyde	*B*. *papuana*, *B*. *japonica*	[9,25]
**74**	ferulyl aldehyde-β-D-glucoside	*B*. *japonica*	[25]
**75**	methyl cinnamate	*B*. *polyandra*, *B*. *fungosa*	[16,31]
**76**	cinnamic acid	*B*. *polyandra*, *B*. *abbreviata*	[16,26]
**77**	coniferyl aldehyde	*B*. *abbreviata*	[26]
**78**	methyl caffeate	*B*. *harlandii*	[1]
**79**	cinnamoyl-β-D-glucopyranose	*B*. *polyandra*	[16,31]
**80**	*p*-hydroxycinnamoyl-β-D-glucopyranose	*B*. *polyandra*, *B*. *laxiflora*	[16]
**81**	4'-hydroxy-3'-methoxycinnamoyl-β-D-glucopyranose	*B*. *polyandra*	[16]
**82**	balaxiflorin B = (6'-*O*-(*E*)-caffeoyl coniferin)	*B*. *laxiflora*	[5]
**83**	2-*O*-(*E*)-caffeoyl-1-*O*-*p*-(*E*)-coumaroyl-β-D-glucopyranose	*B*. *japonica*	[14]
**84**	1-*O*-(*E*)-caffeoyl-β-D-glucopyranose	*B*. *laxiflora*, *B*. *harlandii*, *B*. *polyandra*, *B*. *laxiflora*	[1,5,7,14-16,20,28]
**85**	1-*O*-(*E*)-caffeoyl-β-gentiobiose	*B*. *japonica*, *B*. *polyandra*	[14,15]
**86**	1-*O*-(3'-*O*-*β*-D-glucopyranosyl)-(*E*)-caffeoyl-β-D-glucopyranose	*B*. *japonica*	[14]
**87**	balajaponin B	*B*. *japonica*	[32]
**88**	balajaponin C	*B*. *japonica*	[32]
**89**	balajaponin E	*B*. *japonica*	[32]
**90**	balajaponin D	*B*. *japonica*	[32]
**91**	isolariciresinol	*B*. *laxiflora*, *B*. *abbreviata*	[5,26]
**92**	isolariciresinol-4-*O*-β-D-glucoside	*B*. *laxiflora*, *B*. *polyandra*, *B*. *abbreviata*	[5,15,26]
**93**	(+)-pinoresinol-di-*O*-β-D-glucopyranoside	*B*. *spicata*, *B*. *japonica*	[12,21]
**94**	(+)-pinoresinol-*O*-β-D-glucopyranoside	*B*. *laxiflora*, *B*. *japonica*	[5,21]
**95**	(+)-pinoresinol	*B*. *polyandra*, *B*. *abbreviata*	[16,26,33]
**96**	(−)-pinoresinol	*B*. *harlandii*, *B*. *papuana*, *B*. *abbreviata*	[7,9,26]
**97**	(−)-pinoresinol-*β*-D-glucoside	*B*. *japonica*, *B*. *abbreviata*	[25,26]
**98**	balaxiflorin A = (7'*S*, 8*R*, 8'*R*)-9-*O*-galloyllariciresinol-4'-*O*-β-D-glucopyranoside	*B*. *laxiflora*	[5]
**99**	lariciresinol-4-*O*-β-D-glucoside	*B*. *polyandra*	[15]
**100**	lariciresinol-4'-*O*-β-D-glucoside	*B*. *laxiflora*, *B*. *polyandra*	[5,15]
**101**	(−)-lariciresinol	*B*. *harlandii*, *B*. *japonica*, *B*. *abbreviata*	[5,7,15,25,26]
**102**	balanophonin	*B*. *japonica*	[25]
**103**	balanophonin 4-*O*-β-D-glucopyranoside	*B*. *japonica*	[21]
**104**	7 *S*, 8*R* dehydrodiconferyl alcohol	*B*. *latisepala*	[30]
**105**	3,3'-bis (3,4-dihydro-6-methoxy-2 *H*-1-benzopyran)	*B*. *fungosa*	[16]
**106**	balajaponin A	*B*. *japonica*	[32]
**107**	brevifolin	*B*. *simaoensis*	[28]
**108**	methyl brevifolincarboxylate	*B*. *involucrata*	[34]
**109**	kaempferol	*B*. *spicata*	[12]
**110**	quercetin	*B*. *simaoensis*	[23]
**111**	quercimeritrin	*B*. *simaoensis*	[23]
**112**	3-hydroxyphloretin	*B*. *harlandii*, *B*. *tobiracola*	[1,35]
**113**	hesperetin dihydrochalcone 4'-β-D-glucoside	*B*. *harlandii*, *B*. *tobiracola*	[1,35]
**114**	3-hydroxyphloretin-4'-*O*-β-D-glucoside	*B*. *harlandii*, *B*. *tobiracola*	[1,8,35]
**115**	phloretin 4'-*O*-β-D-glucoside	*B*. *harlandii*, *B*. *tobiracola*	[1,8]
**116**	phloridzin	*B*. *involucrata*	[34]
**117**	3-hydroxy-phloridzin	*B*. *involucrata*	[34]
**118**	(*E*)-3,4,2',4',6'-pentahydroxychalcone-2'-*O*-β-D-glucopyranoside	*B*. *involucrata*	[34]
**119**	aureusidin-4-*O*-β-D-glucopyranoside	*B*. *involucrata*	[34]
**120**	catechin	*B*. *spicata*	[19]
**121**	(2*R*/2 *S*)-eriodictyol 7-*O*-β-D-glucopyranoside	*B*. *harlandii*, *B*. *tobiracola*, *B*. *involucrata*	[1,8,36]
**122**	(2*R*/2 *S*)-eriodictyol (= 2-(3,4-dihydroxyphenyl)-2,3-dihydro-5,7-dihydroxy-4 *H*-1-benzopyran-4-one)	*B*. *harlandii*, *B*. *involucrata*	[1,36]
**123**	(2*R*/2 *S*)-eriodictyol 5-*O*-β-D-glucopyranoside	*B*. *involucrata*	[36]
**124**	eriodictyol 7-(6''-*O*-galloyl-β-D-glucoside)	*B*. *harlandii*	[1]
**125**	monogynol A	*B*. *spicata*	[19]
**126**	monogynol A 3-palmitate	*B*. *simaoensis*	[12]
**127**	lupenoe	*B*. *spicata*, *B*. *indica*	[19,37]
**128**	lupeol	*B*. *involucrata*	[16,29,37,38]
**129**	lupeol acetate	*B*. *harlandii*, *B*. *polyandra*, *B*. *involucrata*, *B*. *indica*, *B*. *japonica*	[7,16,24,27,29,38,39]
**130**	Balanophorin B	*B*. *spicata*, *B*. *indica*, *B*. *simaoensis*	[19,38,39]
**131**	β-amyrin	*B*. *laxiflora, B*. *involucrata*, *B*. *simaoensis*	[7,29,37,38,40]
**132**	β-amyrin acetate	*B*. *spicata*, *B*. *indica*, *B*. *simaoensis*, *B*. *japonica*	[19,24,39,40]
**133**	Balanophorin A	*B*. *spicata*, *B*. *indica*, *B*. *simaoensis*, *B*. *involucrata*	[19,37-39]
**134**	β-amyrin stearate	*B*. *simaoensis*	[12]
**135**	β-amyrone	*B*. *indica*, *B*. *simaoensis*	[38,39]
**136**	ursa-12-ene-11-one-3-oloctocosate	*B*. *involucrata*	[37]
**137**	taraxastenone	*B*. *abbreviata*	[40]
**138**	taraxasterol	*B*. *abbreviata*	[31]
		*B*. *indica*	
**139**	olean-12-ene-3,11-dione	*B*. *tobiracola*	[41]
**140**	shanzhioside methyl ester	*B*. *spicata*	[12]
**141**	geniposide	*B*. *spicata*	[12]
**142**	loganin 6'-*O*-β-glucopyranoside	*B*. *latisepala*	[30]
**143**	clerosterol	*B*. *harlandii*	[7]
**144**	clerosterol-3-*O*-(6'-*O*-palmitoyl)-β-D-glucopyranoside	*B*. *harlandii*	[7]
**145**	β-sitosterol	*B*. *polyandra*, *B*. *involucrata*, *B*. *simaoensis*, *B*. *japonica*	[16,24,27,29,37,38]
**146**	daucosterol	*B*. *simaoensis*, *B*. *involucrata*	[23,29,37]
**147**	β-sitosterylglucoside-3'-*O*-linoleate	*B*. *involucrata*	[42]
**148**	palmitic acid	*B*. *harlandii*, *B*. *simaoensis*	[7,38,39]
**149**	4-hydroxybenzyl-β-D-glucoside	*B*. *polyandra*	[15]

2.1 *Tannins*. There are abundant and varied tannins (1–61) in the genus *Balanophora*. The hydrolyzable tannins - one and / or several galloyls, HHDP, caffeoyl and coumaroyl groups attached to one glucosyl unit by ester linkage - were reported as predominant components from this genus.

Cinnamic acid derivative tannins were found as characteristic components in this genus. They have a caffeoyl, feruloyl, coumaroyl or cinnamoyl group connected at C(1) position in a glucosyl moiety by acyl *O*-glycosidic linkage. Positions C(3) and C(4) in glucosyl moiety are usually attached to a galloyl, together with a HHDP group often linked to the C(4) and C(6) positions. The C(2) position often has a unsubstituted hydroxyl group. In addition to the aforementioned connection types, the 1, 2-di-, 1, 3-di- and 1, 2, 6-tri-caffeoyl compounds (e.g. compounds 5, 6 and 13), which were also from the genus *Balanophora,* were regarded as cinnamic acid derivative tannins [14,17]. Up to now, compounds 1–13, 28, 30–31, 33 – 41 from *B*. *spicataB*. *fungosaB*. *polyandraB*. *laxifloraB*. *harlandii* and *B*. *japonica* are fall into this category [1,5,7,8,14-21]. To the best of our knowledge, this kind of compounds did not discovered in other families.

The caffeoyl group is connected to the C(1) position in compounds 1–4, 7–12, and the galloyl is linked to the C(3), C(4) and C(6) of the glucosyl moiety. As for the HHDP groups, it was mostly joint to C(4) and C(6) [1,5,7,14,16-18]. Compounds 28–30, isolated from the plants *B*. *polyandra* and *B*. *japonica*, have a caffeoyl or galloyl group at C(1) position and a 1, 1'- (3, 3', 4, 4'-tetrahydroxy) dibenzofurandicarboxyl group attached to the C(4) and C(6) positions of the glucosyl unit [21]. The compounds 31–34 isolated from *B*. *japonica* contain an oxidized-HHDP group named brevifolincarboxylate [22]. For 1-*O*- (*E*) -caffeoyl-6-*O*- (*S*) -brevifolincarboxyl-β-D-glucopyranose (31) and 1, 3-di-*O*-galloyl-6-*O*- (*S*) -brevifolincarboxyl-β-D-glucopyranose (32), their C(6) of glucosyl moiety connect to a brevifolincarboxylate group. The HMBC spectrum and phenylglycine methyl ester (PGME) methods played important roles in the elucidatation of the chemical structures of 31 and 32 [22]. Balanophotannin E (32) exhibited strong cytotoxicity to HepG2 cells, which is equivalent to that of cisplatin. Methylated balanophotannins F (33) by alkaline hydrolysis obtained the heptamethyl derivative. Balanophotannins G (34) has a caffeoyl, 1, 6-diacylated glucopyranosyl and an aromatic lactones structural unit. In particular, the C(2) and C(5) positions in compound 34 present a -COO^-^K^+^ group correspondingly. And the inorganic element K was determined by negative FAB-MS and HR-ESI-MS [22]. Balapolyphorins A (35) was also cinnamic acid derivative tannin with a balapolyphoroyl group at C(6) position in glucosyl unit to form aryl ester and a caffeoyl group to the C(1) by acyl *O*-glycosidic linkage [15]. Compounds 36–41 take the characteristics that a coumaroyl or cinnamoyl is connected to anomeric carbon by *O*-glycosidic bond, and several galloyl and HHDP groups are linked with the C(3), C(4) and / or C(6) positions in glucosyl moiety to form sugar aryl ester linkages [1,5,14,16-18].

Another attended mode was that the OH group at C(1) position in glucosyl unit connected with a galloyl by one galloyl ester glycoside in compounds 14–27, 29 and 42 [1,5,12-15,20,21]. And other positions’ OH groups are substituted by galloyl, HHDP and caffeoyl group. Among them, the C-O (C-3) in 18 and C-6 in 27 are attached to a caffeoyl group. Additional substituent modes, such as 1, 3-di-, 1, 3, 4-tri-, 1, 3, 4, 6-tetra-galloyl groups and 4, 6-HHDP moieties are in existence as well. The balapolyphorins B (42) emerges two galloyl groups in C(1), C(6) and linked a caffeoyl in C(4) [1,5,14,17].

Galloyl, caffeoyl and hexahydroxydiphenoyl esters of dihydrochalcone glucosides (43–51) were found in the plants of *B*. *tobiracolaB*. *harlandii* and *B*. *papuana*[1,8,9]. Compounds 43–48 are structurally characterized by one or ones of caffeoyl, galloyl or HHDP group ester hold to the C(2''), C(3''), C(4'') and C(6'') positions in glucosyl unit of 3-hydroxyphloretin-4'-*O*-β-glucoside [1,8]. Papuabalanols A (50) and papuabalanols B (51) were two new dehydrohexahydroxydiphenoyl (DHHDP) esters of dihydrochalcone glycosides isolated from the ethyl acetate extract of *B*. *papuana*, with the DHHDP skeletons located in C(4'') and C(6'') positions of the glucosyl units [9].

According to the ^1^ H- and ^13^ C-NMR spectrum of compounds 52 – 58, it showed the presence of 1/2 α- and 1/2 β-D-glucopyranosyl units. The duplication (α/β forms) of the signals revealed by glucosyl unit suggested that those compounds were a pair of D-glucosyl anomeric isomers [1,2,14,15].

Furthermore, gallic acid (59) and ellagic acid (61) were the part of hydrolysable tannins [5,7,15,23,24].

2.2 *C*_*6*_*-C*_*3*_*and C*_*6*_*-C*_*3*_*-C*_*6*_*Constituents*. It was reported that about forty-four phenylpropanoids (62–105), three coumarins (106–108) and sixteen flavonoids (109–124) occurred in genus *Balanophora*, the investigated plants included *B*. *simaoensisB*. *spicataB*. *involucrataB*. *japonicaB*. *harlandiiB*. *polyandraB*. *latisepalaB*. *laxifloraB*. *abbreviata* and *B*. *fungosa*[1,5,7,8,15,16,19-21,23-28,30-32,43].

2.2.1 *C*_*6*_*-C*_*3*_*Constituents*. From 1969, varieties of those constituents were gradually reported from the genus *Balanophora*[7]. The isolated phenylpropanoids chiefly ranged over simple phenylpropanoids, lignans and coumarins. Numbers of those compounds is from 63 to 106, with compounds 62–89 are a term of simple phenylacrylic acid derivatives [1,5,7,14-16,19,23,25-29,32,33,44]. Compound 87 have two coniferin molecules and the location of linkage is explicated to be between C(9) and C(9'). Compounds 89 and 90 possess two coniferin units, and balajaponin C (88) is the methylated compound 89’s aglycon. According to the HMBC and ROESY spectra in balajaponin D (90), the linkage of the two units was assigned to be between C(6) and C(9'), while C(8) and C(9') in balajaponin E (89) [32]. The compounds 93–97 were three lignans with a bisepoxy skeleton isolated from *B*. *spicataB*. *abbreviata* and *B*. *japonica*, respectively [5,7,12,16,25,26]. (+) -Pinoresinol-di-*O*-β-D-glucopyranoside (93) is a glycoside of disaccharide lignan [12,21]. Compounds 98–101 are the monoepoxylignan glucosides with the 9-*O*-7' framework, while (−) -lariciresinol (101) occupied the opposite configuration compared with others [5,7,15,25,26]. Balaxiflorin A (98) has a galloyl ester group at C(9) and its C(7), C(9') positions combines an epoxy lignan moiety by an oxygen atom [5]. Balanophonin (102) is a new neo-ligan from the fresh above- and under-ground parts in methanol extract’s water-soluble fraction of *B*. *japonica*[25]. 3, 3-bis (3, 4-dihydro-6-methoxy-2 *H*-1-benzopyran) (105) was from the rhizome of the plant *B*. *fungosa*[16].

Compounds 106–108 are coumarins [28,32,34]. Balajaponin A (106) contains a dihydroisocoumarin fragment and one 1, 2, 4-trisubstituted benzene ring based on spectrogram. It was a novel carbon skeleton and some components with this skeleton had been synthesized as pharmaceutical intermediates [32]. The chemical structure of methyl brevifolincarboxylate (108) was illuminated based on the analysis of solid-state NMR, and it received considerable interest mainly because of its strong antiviral properties [34].

2.2.2 *Flavonoids*. Types and quantities of flavonoids and flavonoid glycosides in *Balanophora* are relatively few (109–124) [1,8,12,19,20,24,35,36,39]. So far, the isolated flavonoids from *Balanophora* mainly contain flavonol, flavonone, flavanonol, dihydrochalcone and aurone. From 112 to 118 are seven dihydrochalcone compounds and they showed strong inhibitory effects on α-glucosidase [1,8,35,36]. Aureusidin-4-*O*-β-D-glucopyranoside (119) in *B*. *involucrata* was the only aurone from the genus *Balanophora*[34].

2.3 *Terpenoids*. Some pentacyclic triterpenoids (125–139) and three iridoids (140–142) were found in the genus *Balanophora*[12,19,24,31,37-41,45,46]. The investigated plants are mainly *B*. *spicataB*. *simaoensisB*. *involucrataB*. *japonica* and *B*. *tobiracola*. The categories of pentacyclic triterpenoid contain lupinane, oleanane-type and ursane [7,19,37,39,45]. Balanophorin A (133) and balanophorin B (130) were two terpenoids ingredients reported from *Balanophora* by *Ultee* in 1926 for the first time [38].

2.4 *Sterosl*. Five sterols (143–147) were isolated from the whole plants of *B*. *harlandii* and *B*. *involucrata*[7,12,16,23,27,29,37,38,42,47].

2.5 *Others*. A palmitic acid (148) was isolated from *B*. *simaoensis*, and in *B*. *polyandra* 4-hydroxybenzyl-β-D-glucoside (149) was found [7,15,38,39].

## Biological activities of genus *Balanophora*

In general, the extracts and the isolated compounds of the genus *Balanophora* have remarkable free radical scavenging activities, which is the most notable and important pharmacological activities [1,5,10,15,48]. Beyond that, they also display inhibiting HIV, hypoglycemic, alcoholic sobering and some other effects [17,20,49-52].

3.1 *Radical Scavenging Activities*. Many studies had verified that lots of compounds from this genus were effective anti-oxidants, and the hydrolysable tannins exhibited higher activities than that of other compounds [1,5,15]. Especially, the compounds with more adjacent phenolic OH groups (galloyl, pyrogallol, or catechol group) had higher DPPH radical-scavenging activities [5,15].

*She et al*. reported the acetone extract of the fresh female plant of *B*. *laxiflora* had obviously radical scavenging activity in DPPH assay (*IC*_50_ = 16.4 μg/ml). All the isolated compounds (8–10, 13–15, 53–54, 59, 70, 82, 91–92, 94, 98 and 100) from the plants were evaluated the free-radical-scavenging activities by DPPH assay with ascorbic acid as positive control. In this assay, the hydrolysable tannins presented higher activities than the other kinds of the phenolic compounds, and the balaxiflorin A (98) and balaxiflorin B (82), with galloyl or caffeoyl groups attached, showed stronger activity than matching phenol compounds [5]. *Ho* and others convinced the *IC*_50_ values of 6.0, 3.0, 6.4, and 15.7 μg/ml of the crude extract, its derived soluble fractions, EtOAc fraction, BuOH fraction, and water fraction from male flowers of aforementioned plant in DPPH test, (+) -catechin as the reference control. And their *IC*_50_ values were 5.4, 4.1, 5.8, 20.4 μg/ml in superoxide radical scavenging assay (NBT assay), while the *IC*_50_ of (+)-catechin is 9.0 μg/ml. Meanwhile, the methanolic extract of the male flower showed stronger DPPH radical scavenging activity and superoxide radical scavenging assay than that of female flower’s extract [20].

The free radical scavenging rate of 4 mg/ml crude extract of *B*. *involucrata* was 94.34%. *Wang et al*. reported that the 80% acetone extract of *B*. *polyandra* displayed high free radical scavenging activity with *IC*_50_ = 14.48 μg/ml [15]. Then, bioassay-guided chemical investigation of the crude extract led to the isolation of 22 phenolic compounds, and they showed striking radical-scavenging activities. The dihydrochalcones 44, 45, 47, 112 and 114, which were found from the 80% watery acetone extract from the fresh rhizome of *B*. *harlandii*, with a catechol (= benzene-1,2-diol) moiety as ring B exhibited higher activities than ascorbic acid [1,52].

DPPH free radical scavenging assay and ferric reducing power assay of the plant *B*. *spicata* by *Deng et al*. revealed that the ethyl acetate extract, *n*-butanol extract, ethanol extract and water-soluble crude polysaccharides had similar anti-oxidatant activities compared with vitamin *C*. The DPPH experiment (18.91%) indicated that the scavenging rate was relatively stronger than that of vitamin *C* (13.25%) while the content of ethyl acetate extract was lower than 0.02 mg/ml. When the content of water fraction was 0.06 mg/ml, the scavenging rate of it was 91.08%, which closed to the 95.44% of vitamin *C*. The *IC*_50_ of *B*. *spicata* ethyl acetate extracts was 6.0 μg/ml, while the chloroform extract was the highest [10].

3.2 *Inhibit HIV Effects*. 1, 2, 6-tri-*O*-caffeoyl-β-D-glucopyranose (TCGP) (**13**) and 1, 3-di-*O*-caffeoyl-4-*O*-galloyl-glucopyranose (DCGGP) (**11**) were in a dose-dependent relationship in inhibiting the entry of HIV-1 Env pseudovirus into the target cells, with *IC*_50_ values of (5.5 ± 0.2) and (5.3 ± 0.1) μg/ml. The result showed that TCGP and DCGGP were potent HIV-1 entry inhibitors targeting gp41 and could serve as lead ingredients for developing the novel anti-HIV-1 microbicides for prevention of sexual HIV-1 transmission [17]. *Sun et al*. has convinced that compound 1, 2, 6-tri-*O*-galloyl-β-D-glucopyranose (TGGP) (**22**) from *B*. *japonica* could inhibit the form of HIV gp41 six-helix bundle formation with an *IC*_50_ values of (1.37 ± 0.19) μg/ml. And it inhibited gp41-mediated HIV envelope fusion with target cell membrance. The inhibitory activity of TGGP on HIV envelope grlycoprotein-induced cell-cell fusion was detected using a non-infectious cell-based assay [48].

3.3 *Hypoglycemic Effects*. The literature 50 showed that the 95% ethanol extract of *B*. *polyandra* could decrease both the fasting and no fasting blood glucose concentrations of ICR mice. The glucose tolerance was improved remarkably in both normal and alloxan-induced diabetic mice. Compared with normal mice, the blood glucose peak of the ethanol extract of *B*. *polyandra* processed retroposited and declined 40%, the area under the blood glucose-time curve (AUC) declined about 26%. Trace it to its cause, the inhibition of *α*-glucosidase might be one of its major mechanisms [6,49].

3.4 *Anti-inflammatory and Analgesic Effects*. Literatures about anti-inflammatory and analgesic effects of plant *Balanophora* are few. The methanol extracts of *B*. *involucrata* had significant anti-inflammatory and analgesic effect. The *MTD* of it in mice was more than 75 g/(kg·d). Hot plate test indicated the healing effect of it was stronger and better than that of diclofenac - a positive control, and the acting time was more durable than morphine’s. Acetic acid writhing test showed that both the two doses (20 g/kg, 12.5 g/kg) could reduce the number of writhing mice (*P* < 0.01) with the analgesic rates 46.9% and 39.4%. Meanwhile, it could prolong the time the writhing appeared. According to the result of the experiment, the effect of it was equal to the drug hydrocortisone [42,47].

3.5 *Others*. *Jiang et al*. reported that the hydrolyzable tannins 1-*O*- (*E*) -caffeoyl-6-*O*- (*S*) -brevifolincarboxyl-β-D-glucopyranose (31) from *B*. *japonica* with cytotoxicity to HepG2 cancer cells with *IC*_50_ values of 4.22 μg/ml [22]. *Hosoya et al*. found that papuabalanols A (50) showed moderate vasodilator effect on rat aorta, while papuabalanols B (51) had potent inhibition effect of mushroom tyrosinase and anti-melanogenesis in B16 mouse melanoma cells [9]. The (−) -pinoresinol from *B*. *abbreviata* had inhibitory activities on the lipopolysaccharide (LPS)-induced inducible nitric oxide synthase (iNOS) expression in RAW 246.7 cells [31]. And extract of *B*. *polyandra* could improve the high-fat-diet-induced metabolic syndrome by inhibiting the activity of enzyme PTP1B in mice [13].

## Conclusions

The plants of genus *Balanophora* have been used as traditional herbal medicines by local people in China. Herein, we systematically expound the multiplex chemical constructions isolated from plants *B*. *simaoensis*, *B*. *polyandra*, *B*. *spicata*, *B*. *fungosa*, *B*. *harlandii*, *B*. *japonica*, *B*. *laxiflora*, *B*. *tobiracola*, *B*. *papuana*, *B*. *abbreviata*, *B*. *latisepala*, *B*. *involucrata* and *B*. *indica*. Studies indicated that the chemical groups of genus *Balanophora* (Balanophoraceae) were mainly hydrolysable tannins. Cinnamic acid derivative tannins, reported from this genus firstly as a class of novel compounds, are characteristic components in the genus *Balanophora*. Some polymers of hydrolysable tannin and dihydrochalcone glucoside were reported from *B*. *tobiracola*, *B*. *harlandii* and *B*. *papuana*.

The genus *Rhopalocnemis* (family Balanophoraceae) possesses the condensed tannins without presence of hydrolysable tannin [53]. Those remarkable chemical characteristic of aforementioned in two genera could provide evidence for their systematic botany [54].

Additionally, oxidative stress has contributed to the development of many human diseases. The use of antioxidants will be a feasible strategy for the treatment of these diseases. And a good many studies have confirmed that the extracts of *Balanophora* and the isolated compounds possess significant free radical scavenging activities. Some other components still showed the abilities to inhibit HIV and cytotoxicity to cancer cells.

So further studies are necessary to point the chem-diversity and biological significance of these compounds and extend the use of the plants from the genus *Balanophora*.

## Competing interests

The authors declare that they have no competing interests.

## Authors' contributions

XW, ZL, WQ, GS and BL have all been involed in drafting this review. All authors read and approved the final manuscript.

## Supplementary Material

Additional file 1 **The chemical structure of compounds isolated from plants of genus *Balanophora*.** Click here for file
