# Supplementary material for: Phytochemicals and biological studies of plants from the genus Balanophora
Source: Chem Cent J. 2012 Aug 1;6:79. doi: 10.1186/1752-153X-6-79 (PMC3475005; doi:10.1186/1752-153X-6-79)
Supplement: Additional file 1 — The chemical structure of compounds isolated from plants of genus Balanophora . [file 1752-153X-6-79-S1.doc]

**Figure** The chemical structure of compounds that isolated from plants of genus *Balanophora*.
